# Transient transaminitis during an olanzapine to risperidone switch

**DOI:** 10.1192/j.eurpsy.2026.11775

**Published:** 2026-07-31

**Authors:** G. Kontogiannis, N. Kokras

**Affiliations:** First Department of Psychiatry, Eginition Hospital, National and Kapodistrian University of Athens, Athens, Greece

## Abstract

**Introduction:**

Olanzapine and risperidone are second generation antipsychotics, which are very commonly used to treat schizophrenia. Whereas both antipsychotics may rarely cause elevation of aspartate (AST) and alanine (ALT) transaminase, evidence suggests that is relatively more common with olanzapine than with risperidone. However, it is also known that the incidence of adverse drug reactions is further increased by polypharmacy.

**Objectives:**

We report a case of an adult male patient treated with olanzapine and unremarkable baseline AST and ALT levels while treated with olanzapine monotherapy, who developed transaminitis while his treatment was cross-titrated to risperidone.

**Methods:**

A 23-years-old male patient with a diagnosis of schizophrenia was involuntary admitted due to an exacerbation of the disease after the patient discontinued his previous antipsychotic treatment (olanzapine 20mg daily). Upon admission, treatment with olanzapine was promptly recommenced, at the previously established dose. However, 15 days later, there was no response to this antipsychotic treatment, therefore a switch to another antipsychotic (risperidone) was decided.

**Results:**

A cross-titration strategy was implemented, as it was thought the safest strategy, and it is depicted in Table 1. Already from the fist days of olanzapine-risperidone co-administration, an AST and ALT elevation was progressively observed, that culminated between the 22nd and 25th day of hospitalization. Although olanzapine was more propable to cause transaminitis, in this case the co-administration of both olanzapine and risperidone was deemed responsible. Therefore, an accelerated cross-titration was implemented towards tha target dose of 6-8 mg risperidone daily (equivalent to 20 mg olanzapine). Remarkably, upon withdrawal of olanzapine, AST and ALT levels normalized rapidly, further confirming that the co-administration was the culprit of the transaminitis. The patient responded well to risperidone treatment, and was discharged a few days later.

**Table 1.** Timeline, antipsychotic dosing and AST/ALT blood levels Table 1. Long description.

Time	Olanzapine (mg daily)	Risperidone (mg daily)	AST (U/L)	ALT (U/L)
Day 1	20	0	26	25
Day 15	20	2	28	40
Day 22	10	4	57	147
Day 25	5	5	54	149
Day 29	0	6	20	62
Day 32	0	6	18	35
Day 39	0	6	15	22

**Conclusions:**

Antipsychotic switching is commonly needed when a patient does not respond adequately. Broadly three switching strategies are used, “direct switch”, “cross-titration”, and “titration and discontinuation”. A cross-titration strategy, with the first drug gradually discontinued and the second drug gradually introduced, is intuitively perceived by many psychiatrists as the safest. However, as evidenced in this case, a direct switch may prevent adverse reactions that are caused or facilitated by the co-administration of two antipsychotic agents.

**Disclosure of Interest:**

None Declared

## Long descriptions

### Table 1. Long description

The table contains five columns: Time, Olanzapine (mg daily), Risperidone (mg daily), A S T (U / L), and A L T (U / L). The data rows are as follows:

* Day 1: Olanzapine 20, Risperidone 0, A S T 26, A L T 25.

* Day 15: Olanzapine 20, Risperidone 2, A S T 28, A L T 40.

* Day 22: Olanzapine 10, Risperidone 4, A S T 57, A L T 147.

* Day 25: Olanzapine 5, Risperidone 5, A S T 54, A L T 149.

* Day 29: Olanzapine 0, Risperidone 6, A S T 20, A L T 62.

* Day 32: Olanzapine 0, Risperidone 6, A S T 18, A L T 35.

* Day 39: Olanzapine 0, Risperidone 6, A S T 15, A L T 22.

The data shows a peak in liver enzymes (A S T and A L T) around Day 22 to Day 25 during the cross-titration of the two medications, followed by a return to baseline levels once Olanzapine was discontinued.

Navigate back to Table 1..

